# Blue-Light Photoactivated Curcumin-Loaded Chitosan Nanoparticles Prepared by Nanoprecipitation and Ionic Gelation: A Promising Approach for Antimicrobial Photodynamic Inactivation

**DOI:** 10.1021/acsabm.5c00200

**Published:** 2025-05-08

**Authors:** Lais Fernandes Aguilera, Leandro Oliveira Araujo, William Marcondes Facchinatto, Regiane Godoy Lima, Montcharles da Silva Pontes, Jhoenne Helena Vasconcelos Pulcherio, Cynthia Suzyelen Albuquerque Caires, Kleber Thiago de Oliveira, Samuel Leite de Oliveira, Anderson Rodrigues Lima Caires

**Affiliations:** † Instituto de Física, Universidade Federal de Mato Grosso do Sul, CP 549, 79070-900 Campo Grande, MS, Brazil; ‡ Departamento de Química, Universidade Federal de São Carlos (UFSCar), Rodovia Washington Luis. km 235—SP-310, CEP 13565-905 São Carlos, SP, Brazil

**Keywords:** curcumin-loaded chitosan nanoparticle, nanoprecipitation and ionic gelation, bacterial inactivation, photodynamic, blue irradiation

## Abstract

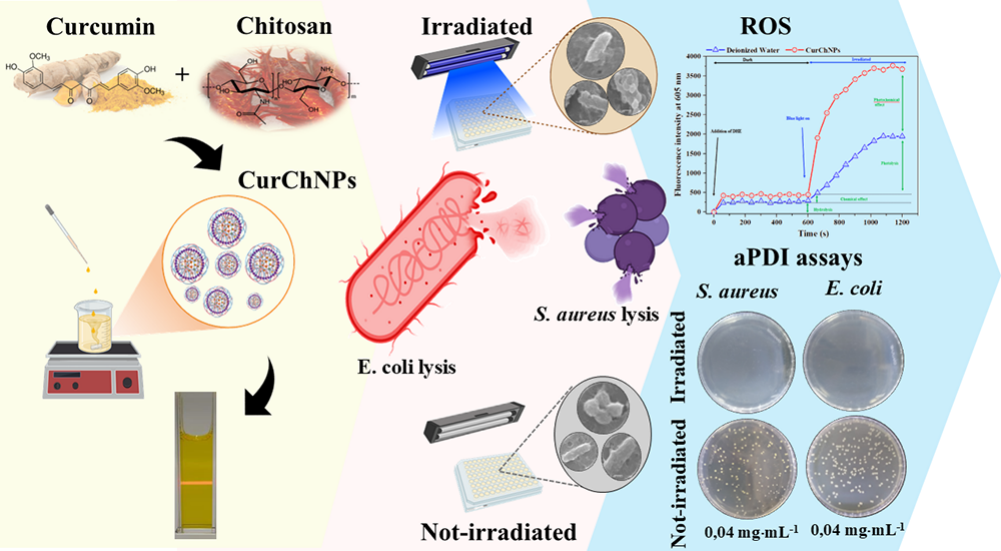

Antimicrobial photodynamic inactivation (aPDI) represents a promising alternative strategy for combating bacterial infections. This study investigates the potential of curcumin-loaded chitosan nanoparticles (CurChNPs) as novel nanoenabled photosensitizer agents for bacterial photoinactivation. CurChNPs were synthesized using an innovative dual synthesis approach by combination of nanoprecipitation and ionic gelation methods; their physicochemical properties were also characterized. The nanoparticles exhibited excellent solubility in aqueous solutions, high curcumin encapsulation efficiency (96%), and controlled release profile. Photoinactivation assays were conducted against *Staphylococcus aureus* (ATCC 25923) and *Escherichia coli* (ATCC 25922) to evaluate the efficacy of CurChNPs in aPDI. The nanoparticles exhibited significant photobactericidal activity when irradiated with blue light (450 nm, 28.84 mW·cm^–2^). Mechanistic studies confirmed the generation of reactive oxygen species (ROS) as the primary mode of photoinactivation. Microscopy analyses revealed structural damage to bacterial cell membranes, culminating in cell lysis. These findings highlight the synergistic effects of the photodynamic activity of curcumin and the antimicrobial activity of chitosan, demonstrating that CurChNPs are a promising platform for the eradication of bacterial infections. This work contributes to the development of sustainable, nanotechnology-based approaches for addressing bacterial infections, particularly against resilient Gram-negative pathogens. Future studies may explore the potential of CurChNPs against antibiotic-resistant bacterial strains.

## 1 Introduction

The emergence of infectious diseases caused by bacterial pathogens represents a significant challenge to global public health. Historical accounts of epidemics such as the Black Death, driven by *Yersinia pestis*, or the cholera pandemics, caused by *Vibrio cholerae*, underscore the devastating impact of bacterial infections.^1−3^ Although antibiotics revolutionized medicine, enabling the treatment of infections once deemed incurable,^4^ their misuse and overuse have catalyzed the rise of antibiotic-resistant bacteria. This growing resistance crisis, combined with stagnation in the development of new antibiotics,^5^ underscores the urgent need for alternative antimicrobial strategies.^6^

One promising approach in the fight against bacterial infections is antimicrobial photodynamic inactivation (aPDI), which involves a photosensitizer (PS), a light source of a specific wavelength, and molecular oxygen to generate reactive oxygen species (ROS).^7^ These species can damage essential cellular structures, destroying microbial cells. Unlike antibiotics, which typically target specific molecular pathways, aPDI multitarget mechanism reduces the likelihood of resistance development.^8^ Additionally, its high selectivity and localized action make aPDI particularly attractive for clinical and environmental applications.^9^

Nanotechnology has further enhanced the aPDI’s efficacy.^10^ Nanoparticles (NPs) ^9^offer unique advantages, such as improved drug delivery, enhanced solubility of hydrophobic agents, and controlled release profiles.^11^ Among various nanomaterials, chitosan-based nanoparticles have garnered significant attention due to their biocompatibility, biodegradability, and inherent antimicrobial properties.^12,13^ Chitosan, a natural polysaccharide derived from chitin, inherently possesses antibacterial, antifungal, and antioxidant activities.^14^ Its ability to interact with bacterial membranes and its versatility in forming nanoparticles make it a valuable candidate for drug delivery and photodynamic applications. The cationic nature of chitosan enables electrostatic interactions with anionic bacterial membranes, facilitating targeted delivery and membrane destabilization.^15−17^ Its unique ability to form stable nanoparticles *via* ionic gelation-driven by protonated amino groups cross-linking with polyanions like tripolyphosphate (TPP), ensures their controlled cargo release and enhanced biofilm penetration. These attributes make chitosan an ideal carrier for photosensitizers, synergistically combining intrinsic antimicrobial activity with photodynamic ROS generation.

Curcumin, a bioactive polyphenolic compound derived from *Curcuma longa*,^18−20^ has been widely studied for its antioxidant, anti-inflammatory, and antimicrobial properties.^21−23^ Moreover, recent research highlights its efficacy against parasites such as *Leishmania braziliensis*, *Leishmania major*, *Toxoplasma gondii*, and *Schistosoma mansoni*, as well as insect vectors of diseases, including mosquitoes and other arthropods.^24−28^ Despite its therapeutic potential, curcumin’s clinical application is limited by its poor aqueous solubility, low chemical stability, and rapid metabolism.^29^ Encapsulating curcumin in nanoparticles, particularly chitosan-based systems, offers a viable strategy to overcome these limitations and biological barriers.^13^ The encapsulation process can improve curcumin’s solubility, stability, and bioavailability, while facilitating its controlled release and targeted delivery.^30^ This synergy is exemplified in recent work where chitosan-curcumin nanocomposites demonstrated enhanced photobactericidal activity against both Gram-positive and Gram-negative pathogens, outperforming free curcumin by 3–5-fold in ROS generation.^31,32^ Such systems leverage chitosan’s mucoadhesive properties to prolong microbial exposure to curcumin’s photodynamic effects, addressing a key limitation of transient ROS production in conventional PDT

In the context of photodynamic therapy, curcumin functions as an effective photosensitizer. The role of curcumin as a photosensitizer date to early studies demonstrating its ability to generate ROS under visible light irradiation.^33,34^ Unlike conventional photosensitizers such as porphyrins or methylene blue, curcumin exhibits intrinsic antimicrobial and anti-inflammatory properties, making it a multifunctional agent for photodynamic applications. Its natural origin and broad absorption spectrum (340–535 nm) have positioned it as a sustainable alternative to synthetic dyes,^33^ with recent advances focusing on nanoparticle encapsulation to overcome solubility limitations.^16^ Upon light activation, it generates ROS, including singlet oxygen and free radicals, capable of inducing oxidative stress in microbial cells.^35^ Recent studies have demonstrated the potential of curcumin-loaded nanoparticles in photodynamic applications,^36,37^ showcasing their ability to enhance antimicrobial efficacy while minimizing off-target effects.

This study aims to address the limitations of free curcumin by synthesizing and characterizing curcumin-loaded chitosan nanoparticles (CurChNPs) for use in aPDI. By leveraging the combined advantages of chitosan and curcumin, the study explores the potential of these nanoparticles as an innovative approach to combat bacterial infections. The nanoparticle preparation process also involves ionic gelation, a method known for producing stable nanoparticles with high encapsulation efficiency. The nanoparticles were extensively characterized using techniques such as atomic force microscopy (AFM), dynamic light scattering (DLS), and Fourier transform infrared (FTIR) spectroscopy to evaluate their physicochemical properties. The study also investigates the biological performance of CurChNPs against *Staphylococcus aureus* (Gram-positive) and *Escherichia coli* (Gram-negative). The evaluation includes their ability to produce ROS upon blue-light activation and their effects on bacterial viability assessed through colony-forming unit assays and electron microscopy.

## 2 Materials and Methods

### 2.1 Materials

Curcumin (≥98%) was synthesized and provided by the Bio-Organic Chemistry Laboratory at the Federal University of São Carlos (UFSCar).^38^ Chitosan, sodium tripolyphosphate (TPP), Poloxamer 407 (P407), and dihydroethidium (DHE) were purchased from Sigma-Aldrich (São Paulo, Brazil). For biological assays, strains of *E. coli* (ATCC 25922) and *S. aureus* (ATCC 25923), Plate Count Agar (PCA), and Brain Heart Infusion Broth (BHIB) (KASVI, São Paulo, Brazil) were used. All chemicals used were of analytical grade.

### 2.2 Preparation of Nanoparticles

The preparation of nanoparticles was carried out following a combination of nanoprecipitation and ionic gelation methods.^31,32^ Initially, 16 mg of chitosan were dispersed in 8 mL of 1% (v·v^–1^) acetic acid and kept under stirring at 0.5 g for 24 h at room temperature, and then the chitosan solution was filtered. After diluting the chitosan, 0.5 g of P407 was added to the chitosan solution under stirring until its complete dissolution. Next, 1 mL of curcumin in tetrahydrofuran (THF, IMPEX) at a concentration of 1.6 mg·mL^–1^ was added drop by drop to initially form nanomicelles of curcumin-P470 (nanoprecipitation) in the presence of chitosan. Finally, 1 mL of TPP (2 mg·mL^–1^) was added drop by drop to obtain curcumin-load chitosan nanoparticles (CurChNPS) *via* ionic gelation. The solution was protected from light and kept under constant stirring at 0.5 g for 12 h to completely evaporate the solvent. After this process, the evaporated volume was replaced by deionized water. Curcumin-free nanoparticles were also produced with chitosan, P407, and TPP prepared at the same concentration as those used for the curcumin-loaded nanoparticles. Additionally, a solution P407 alone diluted in water was prepared at the same concentration as in the nanoparticle formulation.

### 2.3 Size and Morphological Characterization of Nanoparticles

The hydrodynamic size distribution, polydispersity index (PDI), and Zeta potential (ζ) of CurChNPs were measured using dynamic light scattering (DLS) with a Zetasizer Nano-ZS ZEN 3600 (Malvern Instruments Ltd.). Measurements in triplicate were conducted at room temperature on a solution of CurChNPs with a concentration of 0.16 mg·mL^–1^. The Tyndall effect was also evaluated by focusing a red laser beam (633 nm, 5 mW) on the samples, with deionized water as the control.^13^

Morphological images of CurChNPs were collected using an AFM Workshop TT2 atomic force microscope. For this, 10 μL of CurChNPs was deposited onto a silicon substrate and left to dry for 24 h at room temperature. Images were obtained in noncontact (vibrating) mode at room temperature, employing an aluminum-coated silicon cantilever with a spring constant of 5.0 N·m^–1^ and a resonance frequency of 160 kHz. Image processing and adjustments were performed using Gwyddion software (64-bit).

### 2.4 Optical Characterization of Nanoparticles

Ultraviolet–visible (UV–vis) absorption spectrum was recorded in the 250–600 nm range with a bench spectrometer (lambda 265 UV/vis, PerkinElmer). For this, 100 μL of the CurChNPs (0.16 mg·mL^–1^) was diluted to a total volume of 2 mL with deionized water in a quartz cuvette. Fluorescence measurements were performed using a spectrofluorometer (FluoroMate FS-2, Scinco) by exciting the CurChNPs at 460 nm and collecting the emission between 470 and 780 nm. UV–vis absorption and fluorescence were conducted using a 10 mm optical path length quartz cuvette with four polished faces, at room temperature.

CurChNPs and their precursors were also characterized by Fourier transform infrared spectroscopy (FTIR) on a PerkinElmer Spectrum 100 infrared spectrometer equipped with an attenuated total reflectance (ATR) accessory featuring a germanium crystal. The nanoparticles were freeze-dried for analysis, and spectra were recorded in transmittance mode under the following conditions: 10 scans in the 4000–600 cm^–1^ range with a resolution of 4 cm^–1^.

### 2.5 Curcumin Encapsulation Efficiency

The ultrafiltration centrifugation method was used to determine the encapsulation efficiency (*EE*) of curcumin in CurChNPs. A 500 μL aliquot of nanoparticle solution and 500 μL of free curcumin in ethanol were deposited in ultrafiltration devices of 30 kDa regenerated cellulose (Microcon, Amicon). Then, they were centrifuged (Kasvi) at 25 °C for 60 min at 1956.26*g*, and the absorbance values of the filtrates were measured in a UV–vis spectrophotometer (LAMBDA 265 UV/vis). The quantifiable amount that crosses the membrane represents the concentration of curcumin. The *EE* of curcumin was quantified using eq 1 by calculating the difference between the initial concentration of curcumin and the amount remaining after centrifugation.
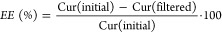
1

### 2.6 *In Vitro* Release Kinetics

The release kinetics of free curcumin and curcumin associated with CurChNPs were evaluated using a two-compartment model. This setup consisted of a donor and an acceptor compartment separated by a cellulose membrane (Spectrapore) with a molecular weight cutoff of 1 kDa. The CurChNPs were placed in the donor compartment, while the recipient compartment was filled with a solvent mixture of 50% water and 50% ethanol, following the same setup used for free curcumin.

Aliquots were collected and analyzed by UV–vis spectroscopy over a 48-h period to determine the percentage of release as a function of time. After analysis, the aliquots were returned to the receiving compartment. All measurements were made in triplicate.

### 2.7 Antimicrobial Photodynamic Inactivation (aPDI) Assays

Gram-positive *S. aureus* (ATCC 25923) and Gram-negative *E. coli* (ATCC 25922) strains were used for aPDI assays. To prepare the bacterial suspensions, 40 μL of the stock culture (kept in the freezer) was added to 5 mL of Brain Heart Infusion (BHI) broth and incubated at 37 °C for 24 h with shaking at 1.12 g. After incubation, the inoculum was diluted in phosphate buffered saline (PBS) solution until turbidity of 1.0 on the McFarland scale.

A 500 μL of bacteria suspension was mixed with 500 μL of nanoparticles diluted in PBS at various concentrations, resulting in 1 mL of solutions containing CurChNPs at 0 (negative control, NC), 0.02, 0.04, and 0.08 mg·mL^–1^, with the bacteria concentration standardized to 1.5·10^8^ CFU·mL^–1^. After that, the samples were internalized for 1 h, and 200 μL aliquot of each sample was distributed in two 96-well microplates separated into two groups: irradiated and nonirradiated (dark). The irradiated group was exposed to blue light (450 nm; 28.82 mW·cm^–2^) for 60 min from LEDs.

A 1:32 dilution was prepared for plating, and 1 μL from each well of this dilution was transferred to the Plate Count Agar (PCA) medium, followed by spreading using the Copacabana method.^39^ After plating, all plates were placed in an incubator at 37 °C for 24 h, and then the colonies forming units were counted. The photoinactivation assays were duplicated, and the statistical analyses were performed using the *t*-Student test (*p* ≤ 0.05) using OriginPro 9 software.

The growth kinetics of *S. aureus* and *E. coli* subjected to CurChNPs were also assessed. After carrying the photoinactivation assays as previously described, 200 μL aliquots from the irradiated and nonirradiated samples (0 and 0.02 mg·mL^–1^) were monitored. Growth kinetics were studied by measuring the optical density (OD) at 650 nm using a microplate reader (BioTek, Synergy H1) for 46 h.

### 2.8 Scanning Electron Microscopy (SEM) of Bacteria

Before the microscopy analysis, the photoinactivation assays were carried out against *S. aureus* and *E. coli* (Section 2.7) using CurChNPs at 0 and 0.08 mg·mL^–1^. After that, 200 μL samples were collected from the irradiated and nonirradiated groups and placed in Eppendorf. To achieve fixation, 1 mL of 2.5% glutaraldehyde in phosphate buffer was added and incubated for 3 h. Subsequently, the samples were centrifuged for 5 min at 78.25*g* at 20 °C. After centrifugation, 1 mL of the supernatant was collected, and 1 mL of phosphate buffer was added; this process was repeated three times. For dehydration, ethanol was used at concentrations of 25, 50, 70, 80, 90 and 100%. The samples were centrifuged for 5 min at 78.25*g* at 20 °C, this process was repeated until the ethanol concentration was 100%.

Next, 10 μL of each sample was deposited onto 18 mm × 18 mm glass coverslips, left to dry, and fixed at room temperature for 24 h. Finally, the samples were mounted on a support and coated with a thin layer of gold using an evaporator (Denton Vacuum Desk III). After gold deposition, the samples were taken to the JEOL model MEV microscope (JSM-6380LV). Images were obtained at a voltage of 15 kV and magnifications of 10,000×, 25,000×, and 50,000×.

### 2.9 Reactive Oxygen Species Generation

The ROS production induced by CurChNPs was determined.^40^ A 0.06 mL of an aqueous solution of CurChNPs (0.1 mg·mL^–1^) was added to 0.14 mL of dihydroethidium (DHE) diluted in deionized water (0.34 mM). A saturating concentration of DHE was used. Emission spectra of the samples were obtained in the 515–700 nm range under excitation at 500 nm using a benchtop fluorimeter (FluoroMate FS-2, Sinco). A quartz cuvette with an optical path of 1 cm and four polished faces was used. The production of ROS related to the CurChNPs was initially evaluated in the dark for 10 min and then the samples were exposed to blue light (450 nm) with an intensity of 28.82 mW·cm^–2^ for 10 min. During this process, a fluorescence spectrum was collected every 1 min. The same procedure was performed with negative control, in which 0.14 mL of DHE diluted in deionized water at 0.34 mM was monitored in the dark for 10 min and under irradiation for 10 min.

## 3 Results and Discussion

### 3.1 Preparation and Characterization of CurChNPs

**Figure 1:**
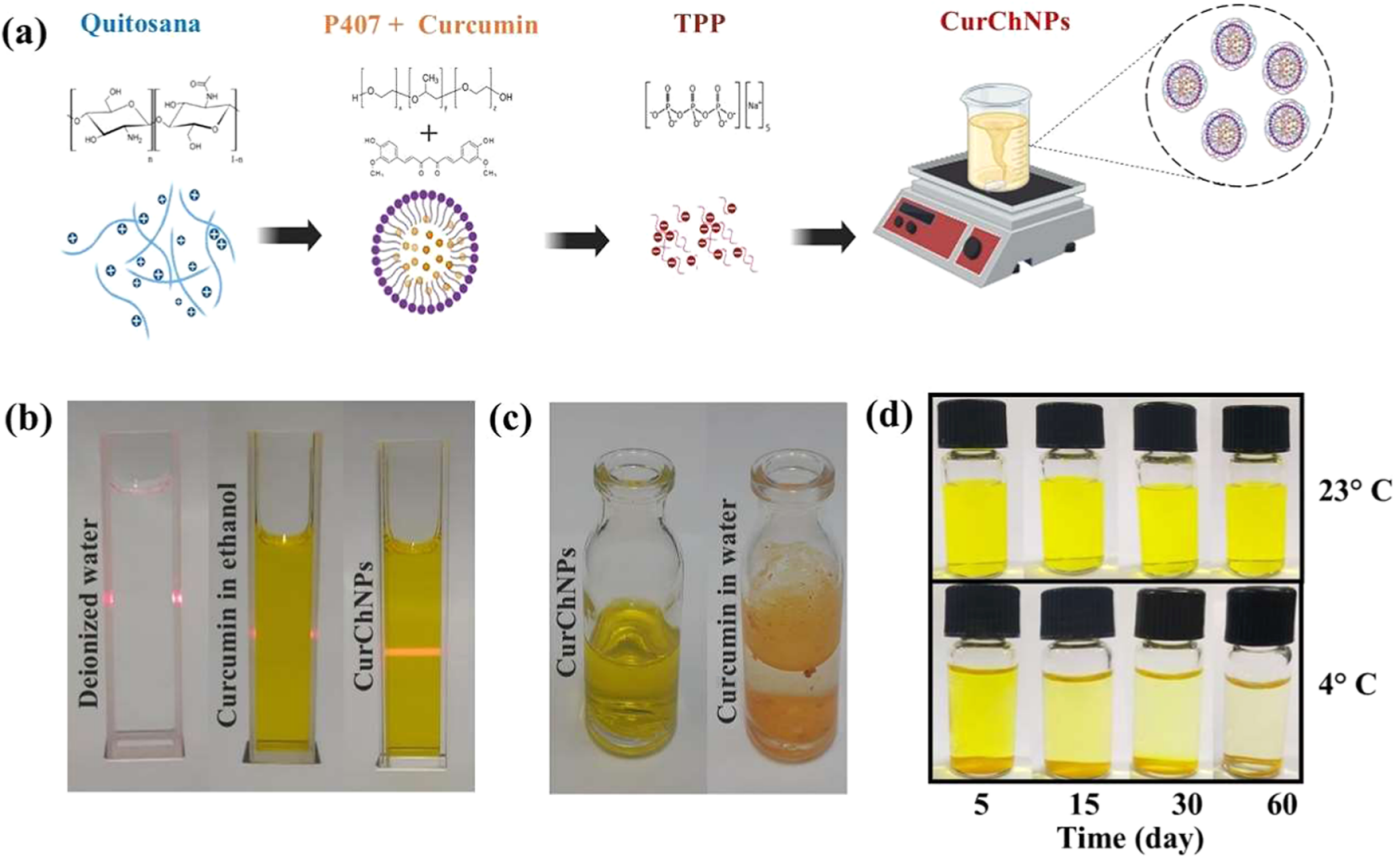
(a) Schematic representation of the formation of CurChNPs; (b) evaluation of the Tyndall effect in the solutions; (c) images of CurChNPs and free curcumin in water, presenting the CurChNPs solubility; (d) images of CurChNPs as a function of storage time under two conditions: room temperature and refrigeration.

CurChNPs nanoparticles were obtained as schematically represented in Figure 1a. P407 acted as a surfactant in the solubilization and stabilization of curcumin in aqueous solution for formation of the nanoparticles (Figure 1a). The curcumin concentration in the CurChNPs was determined as presented in Section S1 of the Supporting Information. The encapsulation efficiency of CurChNPs was high, 96%, pointing to the affinity of curcumin with the polymeric matrices. The direct encapsulation of curcumin using chitosan and TPP *via* ionic gelation results in a low encapsulation efficiency (below 10%), according to Duse et al.^41^ This underscores the significance of incorporating P407 in the approach reported here, which combines nanoprecipitation and ionic gelation methods for CurChNPs preparation. A high encapsulation efficiency of curcumin has been found for different polymer-based nanoparticles.^42,43^ Curcumin-loaded chitosan nanoparticles produced by the flash nanoprecipitation presented an encapsulation efficiency above 95%,^42^ while cellulose nanocrystal nanoparticles carrying curcumin showed an encapsulation efficiency of over 90%.^43^

The Tyndall effect confirmed the formation of a colloidal solution for CurChNPs (Figure 1b), as the light scattering phenomenon occurs when a colloidal solution contains particles smaller than the wavelength of visible light, which ranges from 400 to 800 nm.^44^ No light scattering was observed in the homogeneous and noncolloidal solutions, such as deionized water and curcumin diluted in ethanol (Figure 1b).

The CurChNPs exhibited good solubility in aqueous solution and high stability when stored at room temperature, as shown in Figure 1c,d, respectively. However, nanoparticle precipitation was observed for CurChNPs stored at 4 °C after 15 days of storage at 4 °C, whereas no precipitation was observed even after 60 days of storage at 23 °C (Figure 1d).

**Figure 2:**
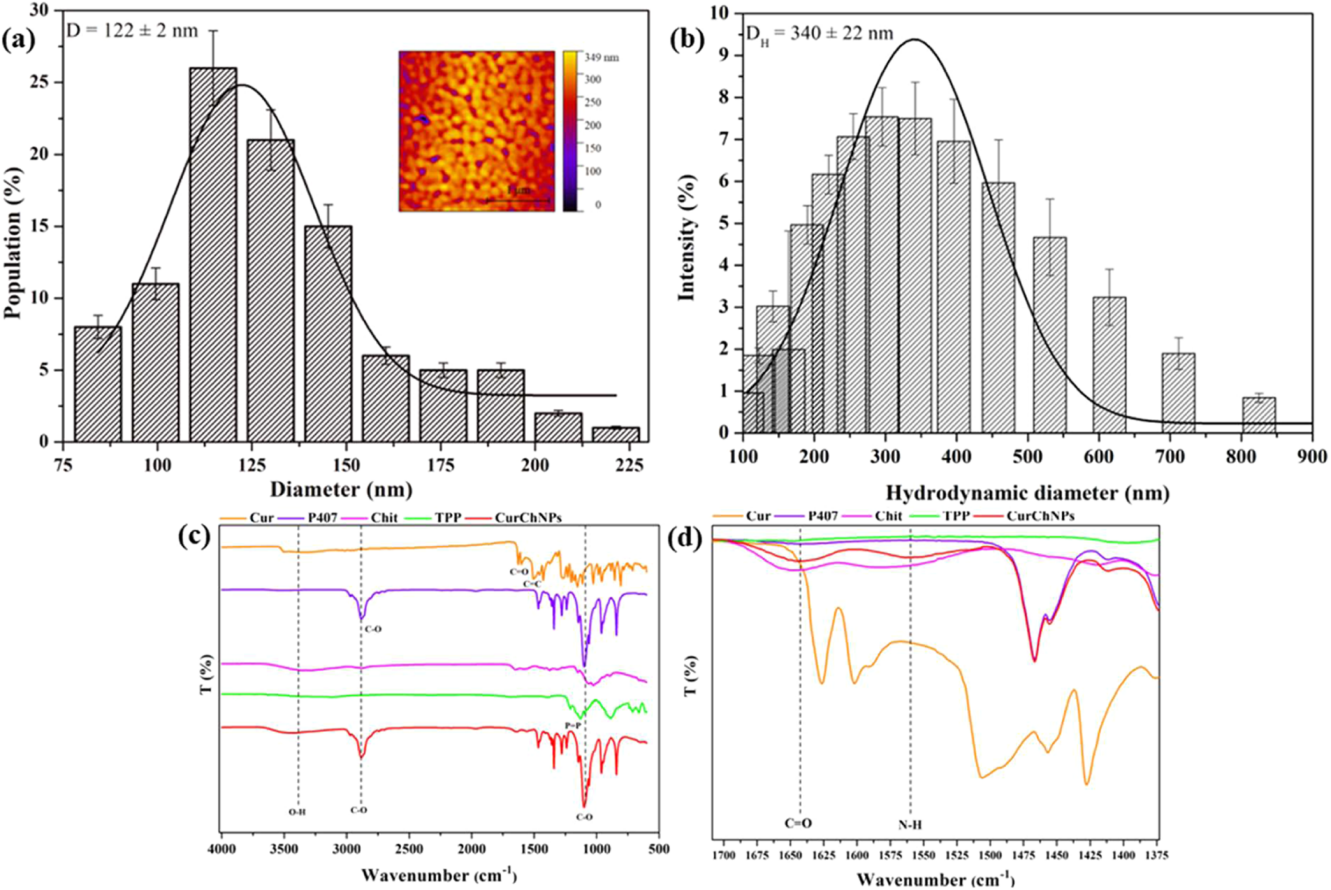
CurChNPs size distribution histogram determined by (a) AFM and (b) DLS, (c) FTIR spectra of CurChNPs and their constituents (c) between 4000 and 500 cm^–1^, and (d) a detailed view of the fingerprint region from 1700 to 1375 cm^–1^.

The AFM results revealed CurChNPs with a mean diameter of 122 ± 2 nm (Figure 2a). Additionally, a larger hydrodynamic diameter of 340 ± 22 nm was determined by the DLS results (Figure 2b), which is in accordance with other curcumin chitosan nanoparticles formulations.^45,46^ For instance, chitosan-curcumin nanoparticles were synthesized for encapsulation in electrospun polycaprolactone (PCL) and gelatin (Gela) structures and nanoparticles with a hydrodynamic diameter of 359 nm were obtained.^45^ In another study, dextran sulfate-chitosan nanoparticles loaded with curcumin with particle sizes in the 180–300 nm range were reported.^46^

FTIR spectroscopy was used to characterize and investigate potential interactions among the CurChNPs constituents (*i.e.*, P407, chitosan, TPP, and curcumin). Figure 2c shows the spectra of the CurChNPs and their constituents. The characteristic bands of free curcumin can be observed at 1629 (C=O), 1605, and 1509 cm^–1^ (C=C), and at 1428 cm^–1^ attributed to C–H stretching in alkenes.^47,48^ These bands reflect important chemical bonds related to the phenol and carbonyl groups involved in the bioactivity.^48^ For P407, the characteristic bands are observed around 1110 cm^–1^ (C–O ether) and 2883 cm^–1^ (C–H), corresponding to the functional groups of this copolymer. The TPP spectrum shows typical bands of the phosphate group (P=O) around 1095 cm^–1^.

For CurChNPs, a shift in the characteristic chitosan band from 3335 cm^–1^ (O–H stretching) to 3456 cm^–1^ indicates an interaction between the copolymer (P407) and chitosan. This alteration can be attributed to the formation of hydrogen bonds or interaction between the hydrophilic groups of chitosan and the hydrophilic blocks of P407—crucial for the stability of the formed micelles and the encapsulation of curcumin. Figure 2d displays a shift of the band from 1572 to 1558 cm^–1^, suggesting an interaction between chitosan and the other nanoparticle constituents, affecting the C=O vibrations in amide I groups of chitosan and the C–N stretching and N–H bending in amide II groups, or the axial deformation of (−NH_2_).^49^ Additionally, curcumin fingerprint bands were not evident in the CurChNPs, which may indicate an efficient encapsulation of curcumin as its typical bands became less intense or, in some cases, disappeared completely. Besides, the P407 profile spectrum remained predominant in the CurChNPs spectrum, reinforcing that curcumin was encapsulated within the micelles formed by this copolymer.

**Figure 3:**
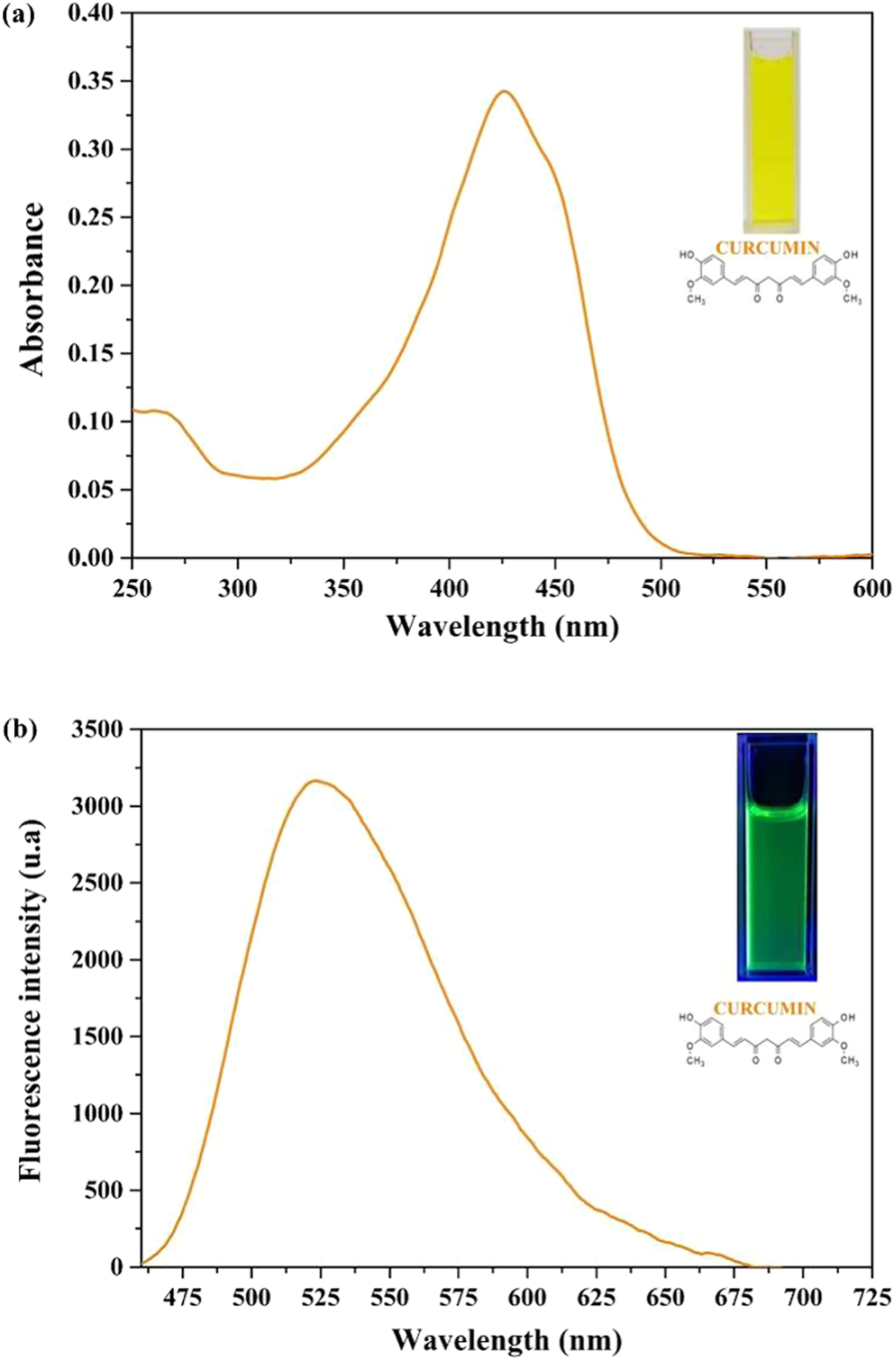
(a) UV–vis absorption and (b) fluorescence spectra of CurChNPs in deionized water. Insets show the visual appearance of CurChNPs under (a) natural and (b) UV light, respectively.

Figure 3 presents the UV–vis absorption and fluorescence spectra of CurChNPs in deionized water. CurChNPs have a maximum absorption of around 425 nm (Figure 3a) and an emission band between 470 and 700 nm when excited at 460 nm, with maximum intensity at around 525 nm (Figure 3b). Both results confirm the characteristic absorption and emission profile of free curcumin (Figure 3a).^50^ The visual appearance of CurChNPs under natural and UV lights is also shown in the insets of Figure 3a,b, respectively.

### 3.2 *In Vitro* Release Kinetics of CurChNPs

**Figure 4:**
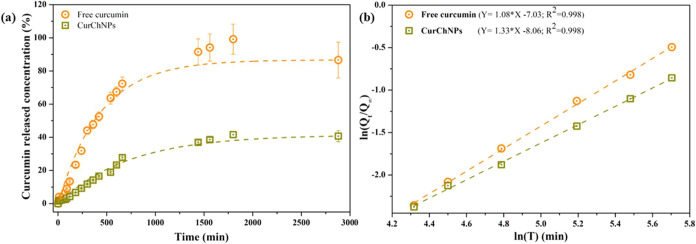
(a) *In vitro* release kinetics of free curcumin and CurChNPs. (b)  as a function of ln(*t*) to determine the release exponent.

A significant difference in the release profile of free curcumin compared to CurChNPs is illustrated in Figure 4a. Free curcumin was rapidly released, with approximately 50% being released within 400 min. In contrast, the nanoparticles demonstrated a slower release, with only about 17% of curcumin released over the same period.

Korsmeyer–Peppas Model (eq 2) adequately describes the observed release kinetics profile (Figure 4b) and provides a better fit to the experimental data, evidenced by a higher *R*^2^ value than other models.
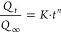
2where *Q*_*t*_ is the amount of curcumin released at a given time *t*, *Q*_∞_ is the amount of curcumin released at infinite time, *K* is the kinetic release constant, and *n* is the release exponent.

The kinetic constants (*K*) for free curcumin and CurChNPs were calculated to be 0.337 and 0.197, respectively. The release exponents (*n*) were also determined to be 1.33 for free curcumin and 1.08 for CurChNPs, indicating a non-Fickian mechanism (super case II). This suggests that the release of CurChNPs involves the dissociation of the polymeric framework. Specifically, the polymeric core of chitosan undergoes disruption, resulting in the release of curcumin. It is worth noting that for the accurate determination of *n*, it is recommended to use experimental data corresponding to less than 60% of the cumulative release .^51−53^ The findings highlight the effectiveness of CurChNPs as controlled release systems for curcumin. Unlike free curcumin, which is released rapidly, nanoparticles proved to be sustained and gradual release due to the polymeric matrices.

### 3.3 Antimicrobial Photodynamic Inactivation (aPDI) Assays

**Figure 5:**
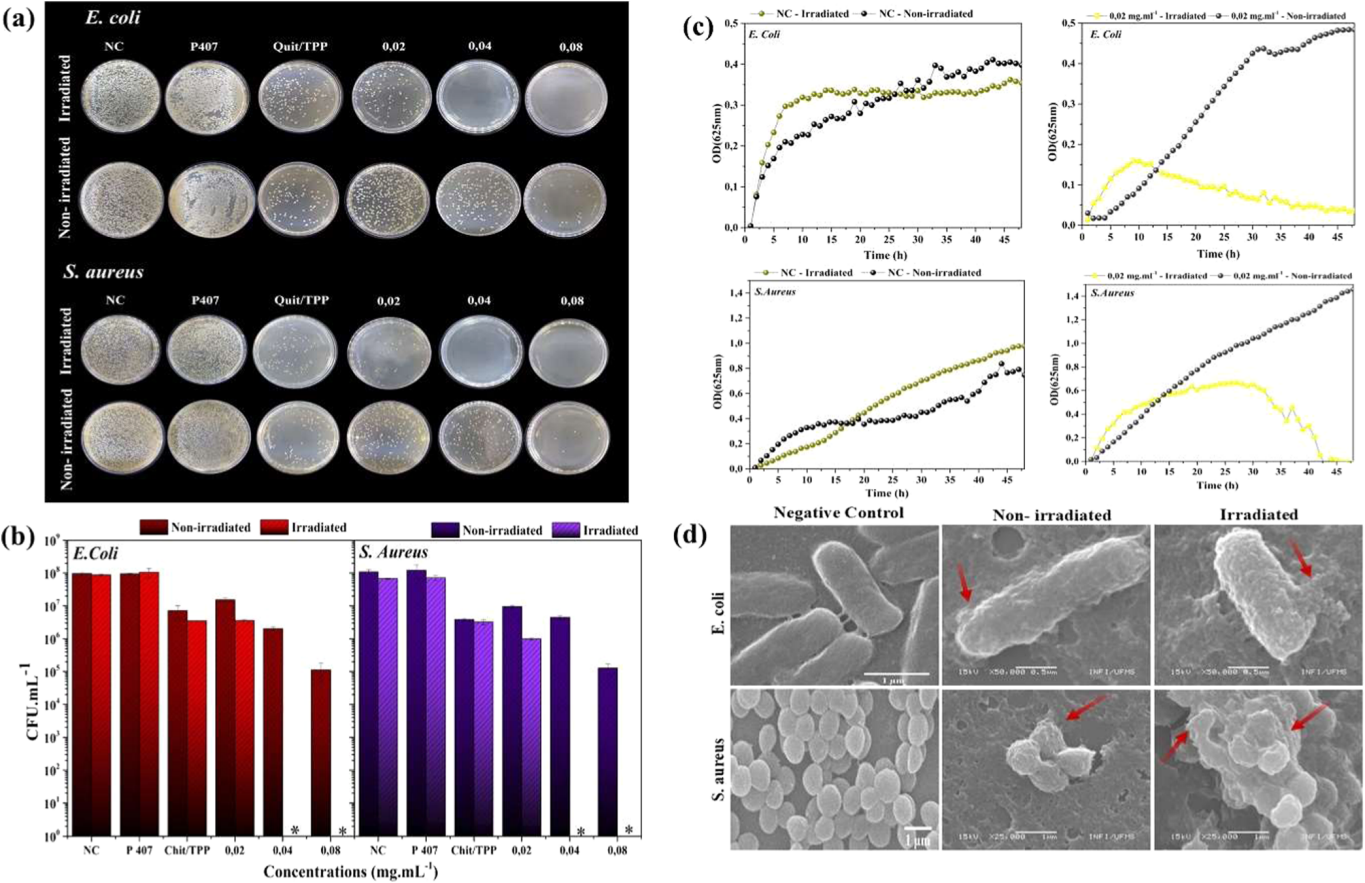
(a) Representative images of CFU growth on Petri dishes. (b) Measured values of CFU·mL^–1^ from aPDI assays against *E. coli* and *S. aureus*. The asterisk (*) denotes photobactericidal activity (>3 log_10_CFU). (c) Bacterial growth kinetics: (c1) *E. coli* negative control; (c2) *E. coli* treated with CurChNPs; (c3) *S. aureus* negative control; and (c4) *S. aureus* treated with CurChNPs. (d) Representative SEM images of *E. coli* and *S. aureus* bacteria after exposure to aPDI assays using CurChNPs at 0.08 mg·mL^–1^.

Figure 5a shows representative images of colony-forming units (CFUs) on Petri dishes for the nonirradiated and irradiated groups in the aPDI assays for the two bacterial strains. CurChNPs exhibited significant photobactericidal activity, resulting in a reduction of more than 3 log_10_CFU at 0.04 and 0.08 mg·mL^–1^ for the irradiated groups across both strains (Figure 5b). Moreover, the Chitosan/TPP solution at 1 mg·mL^–1^ promoted a significant reduction of about 1 log10 compared to the negative control group, even when stored in the dark, attributable to its well-known antimicrobial properties.^54^ Differently, the solution of P407 at 25 mg·mL^–1^ did not show any antimicrobial (chemical) or photoantimicrobial (photodynamic) effects against either bacterial strain.

Figure 5c presents the growth kinetics of *E. coli* and *S. aureus* with and without CurChNPs at 0.02 mg·mL^–1^ and the absence of CurChNPs, during the aPDI process and under dark conditions. The left curves illustrate the growth kinetics of *E. coli* (top) and *S. aureus* (bottom) in the negative control. Irradiated and nonirradiated groups exhibit increased optical density, indicating exponential growth followed by a stationary phase, which reflects a typical bacterial growth curve. The right curves illustrate the growth kinetics of *E. coli* (top) and *S. aureus* (bottom) when exposed to the CurChNPs. The nonirradiated *E. coli* and *S. aureus* display an initial lag phase, followed by a rapid transition into the growth phase and later reaching the stationary phase. In contrast, light-exposed *E. coli* in the presence of CurChNPs show an initial increase in optical density during the exponential phase; however, after about 10 h, there is a marked decline, indicating the onset of the death phase. For *S. aureus*, an exponential increase in optical density was observed when subjected to light and CurChNPs. Still, after about 10 h, it entered the stationary phase, and the death phase initiated after 30 h. These results confirmed that aPDI mediated by CurChNPs is highly effective in restricting bacterial growth and leading to bacterial cell inactivation.

SEM images reveal surface alterations in irradiated bacteria treated with CurChNPs and compared to the control group (Figure 5d). The negative control group displayed *E. coli* and *S. aureus* with smooth and intact surfaces, consistent with their typical morphology. The images of nonirradiated bacteria subjected to CurChNPs showcase the presence of nanoparticles on bacterial membranes, marked by a noticeable increase in surface roughness and evidence of partial membrane damage, indicated by the red arrows. This adherence suppresses bacterial growth due to the bacteriostatic effects characteristic of chitosan,^55^ which is in accordance with results showing that the Chitosan/TPP solution promotes a bacterial growth reduction of ∼1 log_10_. For the irradiated groups, there was a significant increase in surface roughness caused by nanoparticles on the bacterial surface. Additionally, severe bacterial damage is evident, highlighting cell lysis, as indicated by the red arrows. Therefore, SEM analysis confirmed that CurChNPs adhere to the bacterial surface, causing minor membrane damage even in the dark, and completely lysing the cell under blue light irradiation. These membrane disruptions ultimately lead to the death of bacterial cells, aligning with the results of the aPDI assay.

### 3.4 ROS Production by CurChNPs

To establish that the photoinactivation process induced by CurChNPs under blue irradiation is driven by a photodynamic mechanism, the intrinsic ability of CurChNPs to generate reactive oxygen species (ROS) under blue irradiation was evaluated. This was done by monitoring the kinetics of the fluorescent product ethidium, which is formed from the interaction between dihydroethidium (DHE) and ROS promoted by CurChNPs and light, as detailed in Section S2 of the Supporting Information.

**Figure 6:**
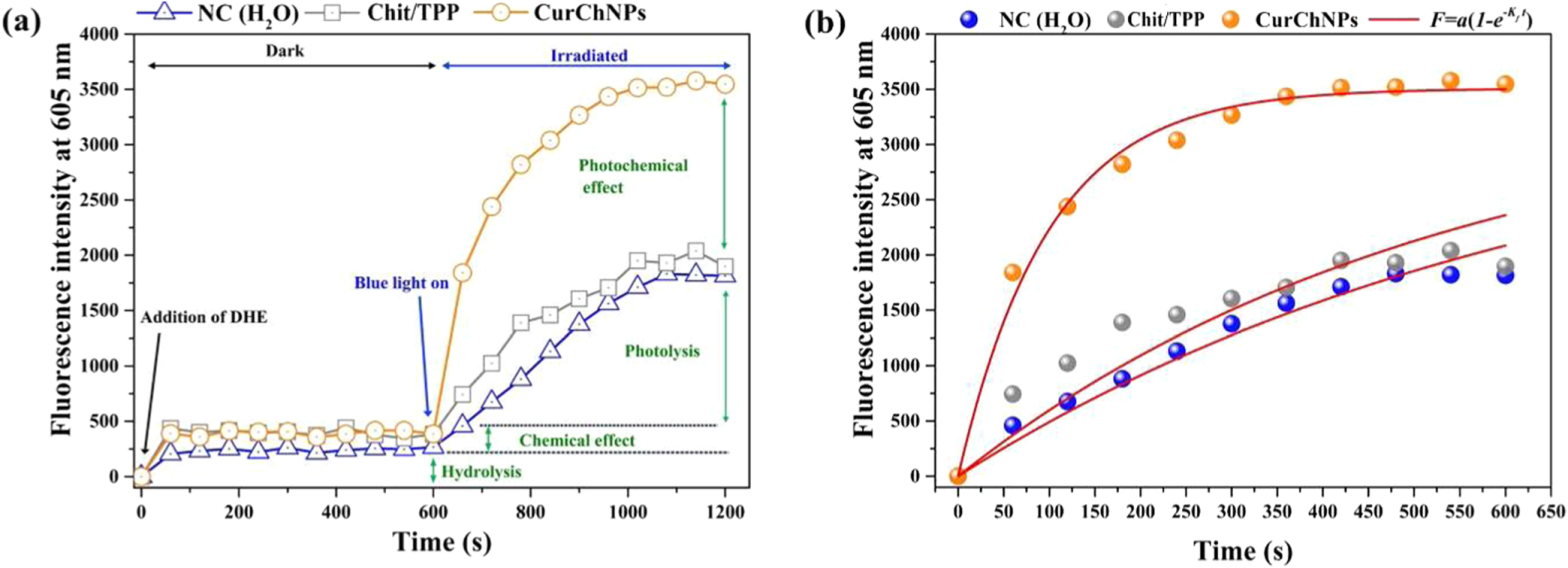
(a) Fluorescence kinetics of ethidium produced by ROS production. (b) Comparison of ROS production by negative control (NC, deionized water), curcumin-free nanoparticles (Chit/TPP), and CurChNPs over irradiation time, along with their corresponding kinetic fittings (red lines).

Figure 6a reveals that the ROS generation capacity of CurChNPs under blue light is significantly higher than that of deionized water (negative control), which generates ROS through photolysis and hydrolysis alone. However, this ROS production by the negative control (H_2_O) and curcumin-free nanoparticles (Chit/TPP) are both insufficient to inhibit bacterial growth, as confirmed by the aPDI assays. This is because Chit/TPP alone does not absorb blue light as can be seen in Figure S3 of the Supporting Information.

The ROS production constant for CurChNPs under irradiation was obtained using eq S3 (Supporting Information) from the fitting of the data shown in Figure 6b, where the constant *a* was fixed, allowing only the constant *k*_f_ to vary. In turn, the ROS production constant (*k*_ROS_) was calculated by dividing *k*_f_ by the DHE concentration (0.34 mM). The calculated *k*_f_ and *k*_ROS_ are shown in Table 1.

**Table 1:** ROS Production Constant of CurChNPs, Chit/TPP and Deionized Water (Negative Control) under Blue Irradiation

sample	*k*_f_ (10^–3^ s^–1^)	*k*_ROS_ (M^–1^ s^–1^)
CurChNPs	10.2 ± 0.5	30.0 ± 1.5
Chit/TPP	1.9 ± 0.2	5.6 ± 0.6
H_2_O (NC)	1.5 ± 0.1	4.4 ± 0.3

The results indicate that the presence of curcumin in the nanoparticle formulation increases the *k*_ROS_ value by approximately 6.8 times compared to when the nanoparticles are absent. Curcumin is widely recognized for its photodynamic activity.^56,57^ Researches have demonstrated its effectiveness, in its isolated form and as part of pharmaceutical formulations, to reduce the growth of pathogenic fungi, including *Sporothrix brasiliensis* and foodborne pathogens like *S. aureus* and *E. coli*. These studies emphasize the photoinactivation mechanisms associated with ROS production.^58,59^

Curcumin-loaded chitosan has shown significant inactivation effects against *S. aureus* and its biofilms on stainless steel surfaces.^60^ Notably, the CurChNPs evaluated in this study exhibited high efficiency in aPDI, underscoring the synergistic effects of chitosan and curcumin. This combined effectiveness results from chitosan’s interaction with bacterial membranes due to its charge, along with the photodynamic activity of curcumin, which has a high capacity for producing ROS. Our findings demonstrate photobactericidal efficacy of CurChNPs against *E. coli* and *S. aureus* highlights their potential as an alternative strategy to combat bacterial infections, including those caused by antibiotic-resistant strains, as supported by recent findings on the role of aPDI in mitigating resistance development.^6,10^

## 4 Conclusions

This study successfully demonstrates the synthesis and characterization of curcumin-loaded chitosan nanoparticles (CurChNPs) as a potent antimicrobial platform for photodynamic inactivation. The innovative combination of nanoprecipitation and ionic gelation yielded nanoparticles with exceptional aqueous solubility, room-temperature stability (>60 days), and a high curcumin encapsulation efficiency of 96%. CurChNPs exhibited controlled release kinetics, with only 17% curcumin released over 400 min, underscoring their potential for sustained therapeutic delivery. The aPDI assays revealed robust photobactericidal activity against both *S. aureus* (Gram-positive) and *E. coli* (Gram-negative), achieving >3 log10 CFU reduction under blue light irradiation (450 nm, 28.84 mW·cm^–2^). Mechanistic studies confirmed ROS generation as the primary inactivation pathway, with CurChNPs producing ROS 6.8× faster than chitosan-only controls. Electron microscopy further validated membrane disruption and cell lysis hypothesis in irradiated bacteria, highlighting the synergistic interplay between chitosan’s intrinsic antimicrobial properties and curcumin’s photodynamic activity. These findings position CurChNPs as a sustainable, nanoenabled alternative to conventional antibiotics, particularly for targeting resilient Gram-negative pathogens. Future work should focus on optimizing formulation scalability and evaluating efficacy against multidrug-resistant strains in preclinical models. The integration of CurChNPs into medical device coatings or topical therapies could be an infection control strategy, addressing critical gaps in current antimicrobial stewardship.

## Supporting Information Available

Calibration curve; kinetic analysis of the ROS production; and absorption spectra of curcumin-free nanoparticles (PDF)
