# Supplementary material for: Blue-Light Photoactivated Curcumin-Loaded Chitosan Nanoparticles Prepared by Nanoprecipitation and Ionic Gelation: A Promising Approach for Antimicrobial Photodynamic Inactivation
Source: ACS Appl Bio Mater. 2025 May 8;8(5):4055–64. doi: 10.1021/acsabm.5c00200 (PMC12093364; doi:10.1021/acsabm.5c00200)
Supplement: Supplementary file 1 — mt5c00200_si_001.pdf [file mt5c00200_si_001.pdf]

Supporting Information

Blue-Light Photoactivated Curcumin-Loaded Chitosan Nanoparticles Prepared by Nanoprecipitation and Ionic Gelation: A Promising Approach for Antimicrobial Photodynamic Inactivation

Lais Fernandes Aguilera[1], Leandro Oliveira Araujo[1], William Marcondes Facchinatto[1], Regiane Godoy Lima[1], Montcharles da Silva Pontes[1], Jhoene Helena Vasconcelos Pulcherio[1], Cynthia Suzyelen Albuquerque Caires[1], Kleber Thiago de Oliveira[2], Samuel Leite de Oliveira[1], Anderson Rodrigues Lima Caires[1] *.

[1]Instituto de Física, Universidade Federal de Mato Grosso do Sul, CP 549, 79070-900 Campo Grande, MS, Brazil

[2]Universidade Federal de São Carlos (UFSCar), Departamento de Química, Rodovia Washington Luis, km 235 - SP-310, São Carlos, SP CEP 13565-905, Brazil.

*Corresponding authors: anderson.caires@ufms.br

Supplementary Sections

S1. Calibration curve

A calibration curve was constructed to quantify the concentration of curcumin in the nanoparticles. Figure S1a shows the absorbance spectra of different concentrations of curcumin in ethanol. The absorbance value at 430 nm as a function of the curcumin concentration for constructing the calibration curve was chosen (Fig. S1b). After CurChNPs production, an aliquot of the nanoparticle solution was dried and then resuspended in ethanol to determine the concentration of curcumin in the nanoparticle formulation using the calibration curve (Fig. S1b).

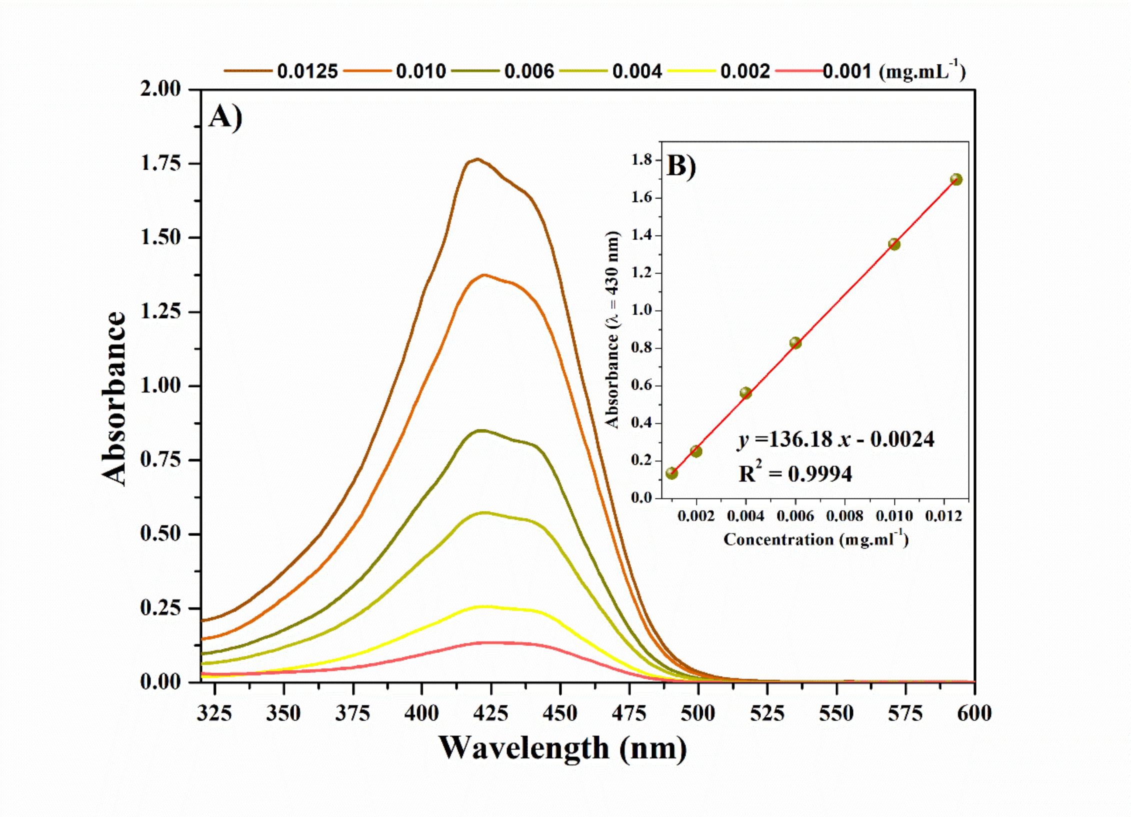

Figure S1: (a) Absorption spectra of curcumin at different concentrations diluted in ethanol; (b) Calibration curve of curcumin at 430 nm.

S2. Kinetic analysis of the ROS production (reaction between DHE and ROS)

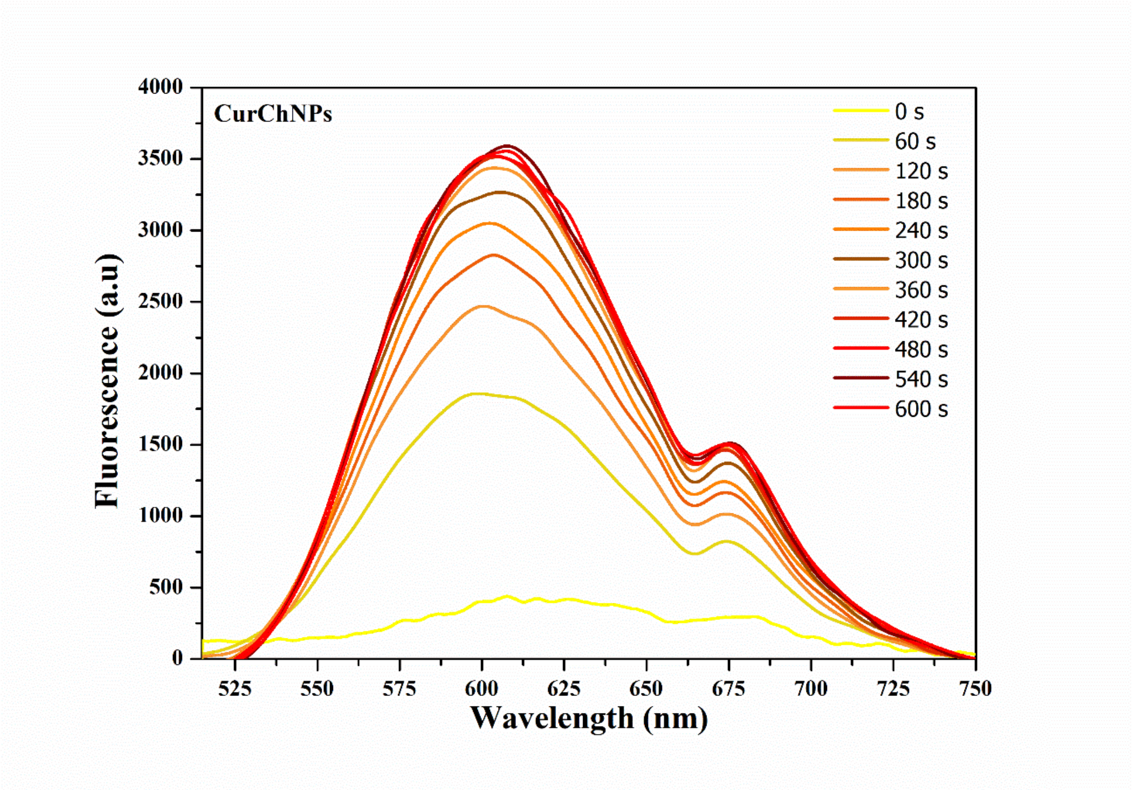

Figure S2: Fluorescence spectra of Ethidium, produced by the interaction between DHE and ROS generated by CurChNPs, over irradiation time.

The rate constant to produce ROS (K_{ROS}), was estimated through kinetic analysis of the fluorescent product generated by the interaction between DHE and ROS. DHE was used at a saturating concentration to ensure that fluorescence measurements did not cease due to insufficient markers for ROS production. It was assumed that the rate of formation of new fluorescent products [F] was proportional to that generated by ROS production due to the CurChNPs under illumination. Therefore, we have:

$$\mathbf{DHE} + \boldsymbol{ROS} \xrightarrow{k} \boldsymbol{F}$$

Consequently, the rate of production of ROS can be written as:

$$-\frac{d[\boldsymbol{ROS}]}{dt} = \boldsymbol{k_{ROS}}\,[\mathbf{DHE}\,][\boldsymbol{ROS}]$$

(S1)

Where (k_{ROS}) is the apparent rate constant for the production of ROS (i.e., the apparent rate constant for the reaction of DHE with ROS), with [ROS] ∝ F. Therefore, Eq. S1 can be rewritten as:

$$-\frac{dF}{dt} = \boldsymbol{k_f}\,\boldsymbol{F} \qquad \text{(S2)}$$

$$\boldsymbol{F} = \boldsymbol{a}(\mathbf{1} - \boldsymbol{e}^{-\boldsymbol{k_f t}})$$

Where, $\boldsymbol{k_f} = \boldsymbol{k_{EROs}}[\mathbf{DHE}\,]$ **:**

$$\boldsymbol{F} = \boldsymbol{a}(\mathbf{1} - \boldsymbol{e}^{-\boldsymbol{k_f t}}) \qquad \text{(S3)}$$

S3. Absorption of curcumin-free nanoparticles (Chit/TPP)

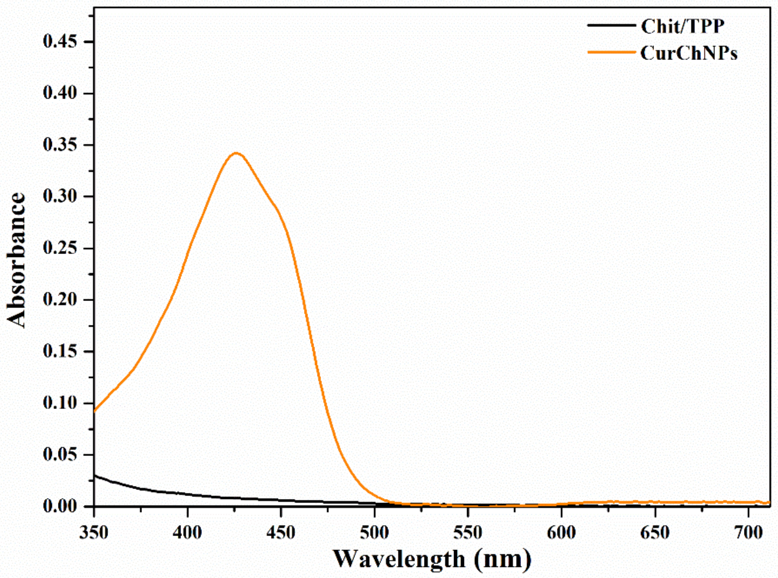

Figure S3: Absorption spectra of curcumin-free nanoparticles (Chit/TPP) and CurChNPs.
